# Prenatal Glucocorticoid Exposure Results in Changes in Gene Transcription and DNA Methylation in the Female Juvenile Guinea Pig Hippocampus Across Three Generations

**DOI:** 10.1038/s41598-019-54456-9

**Published:** 2019-12-03

**Authors:** Andrea Constantinof, Lisa Boureau, Vasilis G. Moisiadis, Alisa Kostaki, Moshe Szyf, Stephen G. Matthews

**Affiliations:** 1 Department of Physiology, University of Toronto, Toronto, ON M5S1A8 Canada; 2 Department of Pharmacology & Therapeutics, Sackler Program for Epigenetics & Psychobiology, McGill University, Montreal, QC H3G1Y6 Canada; 3 Department of Obstetrics and Gynecology, Toronto, Canada; 4 Department of Medicine, University of Toronto, Toronto, ON M5S1A8 Canada; 5 Lunenfeld-Tanenbaum Research Institute, Sinai Health System, Toronto, ON M5G1X5 Canada

**Keywords:** Epigenetic memory, Epigenetics and behaviour, Reproductive biology

## Abstract

Synthetic glucocorticoids (sGC) are administered to women at risk for pre-term delivery, to mature the fetal lung and decrease neonatal morbidity. sGC also profoundly affect the fetal brain. The hippocampus expresses high levels of glucocorticoid (GR) and mineralocorticoid receptor (MR), and its development is affected by elevated fetal glucocorticoid levels. Antenatal sGC results in neuroendocrine and behavioral changes that persist in three generations of female guinea pig offspring of the paternal lineage. We hypothesized that antenatal sGC results in transgenerational changes in gene expression that correlate with changes in DNA methylation. We used RNASeq and capture probe bisulfite sequencing to investigate the transcriptomic and epigenomic effects of antenatal sGC exposure in the hippocampus of three generations of juvenile female offspring from the paternal lineage. Antenatal sGC exposure (F_0_ pregnancy) resulted in generation-specific changes in hippocampal gene transcription and DNA methylation. Significant changes in individual CpG methylation occurred in RNApol II binding regions of small non-coding RNA (snRNA) genes, which implicates alternative splicing as a mechanism involved in transgenerational transmission of the effects of antenatal sGC. This study provides novel perspectives on the mechanisms involved in transgenerational transmission and highlights the importance of human studies to determine the longer-term effects of antenatal sGC on hippocampal-related function.

## Introduction

The hippocampus is critical for many higher brain functions, including learning and memory and regulation of stress responsiveness. In the human, the fetal hippocampus begins to develop at 13–14 weeks, resembling the adult hippocampus by 18–20 weeks of gestation^1^. The mammalian brain, particularly the hippocampus, is highly sensitive to the fetal-maternal environment. The hippocampus has the highest concentration of glucocorticoid receptors (GR) in the brain^2^, and is therefore highly sensitive to the effects of glucocorticoids^3^.

We and others have shown that prenatal exposure to excess glucocorticoids in mice, rats and guinea pigs can alter gene expression related to synaptic plasticity^4^, DNA methylation machinery^5,6^, dopaminergic^7^, serotonergic^4^, and glutamatergic signaling^4,7^, and affects the expression of GR and mineralocorticoid receptors (MR) in the hippocampus^6,8–14^. Altered hippocampal gene expression has been associated with modified regulation of the hypothalamic-pituitary-adrenal (HPA) axis, leading to altered stress responsiveness, and mood and anxiety disorders in animals^15^ and in humans^16^.

Fetal plasma glucocorticoid is maintained at low levels through the majority of pregnancy; however, there is a natural ‘surge’ in fetal glucocorticoid levels at the end of gestation in most mammalian species. This surge is important for maturation of fetal organs, including the lung, kidney and brain^17^. Various factors such as maternal anxiety and stress and placental dysfunction can increase fetal glucocorticoid exposure^18,19^. In addition, the fetus can be exposed to synthetic glucocorticoids (sGC), which are administered to pregnant women at risk for pre-term delivery (~11% of all pregnancies). This treatment promotes lung maturation and reduces morbidity and mortality associated with respiratory distress syndrome in preterm infants^20^. Several studies have demonstrated that exposure of the fetus to high levels of glucocorticoid, prior to the natural glucocorticoid surge, can lead to long-term programming effects on neurocognitive, behavioural, endocrine, and cardiometabolic function^5,8,17,21–23^.

The mechanisms by which premature exposure to elevated levels of glucocorticoids program hippocampal development are poorly understood. Emerging evidence suggests these changes may be mediated through epigenetic mechanisms such as DNA methylation or histone modification. DNA methylation regulates chromatin states and is transmitted through cell division^24^. Administration of antenatal sGCs resulted in alterations in histone-3-lysine-9 acetylation in gene promoter regions^5^ and global DNA methylation changes in fetal organ systems that occurred in first- and persisted to second-generation offspring^25^. In the fetal hippocampus, sGC exposure resulted in acute demethylation of promotor regions 24 h after final exposure. However, promotor hypermethylation in a completely different set of genes was identified 14 days after the last exposure to sGC, as well as major changes in gene expression^6^. These studies suggest that prenatal sGC exposure causes dynamic epigenetic changes which may affect both DNA and histone modifications^5^. We have recently shown that antenatal sGC exposure results in transgenerational changes in behavior and gene expression in the paraventricular nucleus (PVN) of the hypothalamus and medial prefrontal cortex (PFC)^21,26^, however transgenerational epigenetic signatures of exposure have yet to be investigated.

Gene expression is regulated by epigenetic modifications and transcription factor binding at promotor regions, CpG islands, and enhancer regions. Generally, promoters and CpG islands with high levels of DNA methylation are not transcriptionally active, while genes with demethylated promoters are expressed^27–29^. However, recent research has shown that enhancer DNA methylation is more closely associated with gene expression in cancer than promoter methylation^24^. Further, correlations of enhancer DNA methylation and expression of developmental genes have been observed during mouse tissue differentiation^30^, as well as in hippocampal tissue^31^.

Determining the relationship between DNA methylation and transcriptional effects following prenatal sGC exposure will help elucidate the mechanisms by which sGC can permanently program neural function over multiple generations^21^. In the present study, we hypothesized that; (1) antenatal sGC induces changes in hippocampal transcriptional and DNA methylation landscapes across three generations of juvenile female offspring from the paternal lineage, as we have previously demonstrated that young females across three generations show the strongest behavioral and neuroendocrine phenotype^21,26^; (2) Modification of DNA methylation signatures in promoter and enhancer regions relate to changes in hippocampal gene expression.

## Results

### Gene transcription

**Figure 1:**
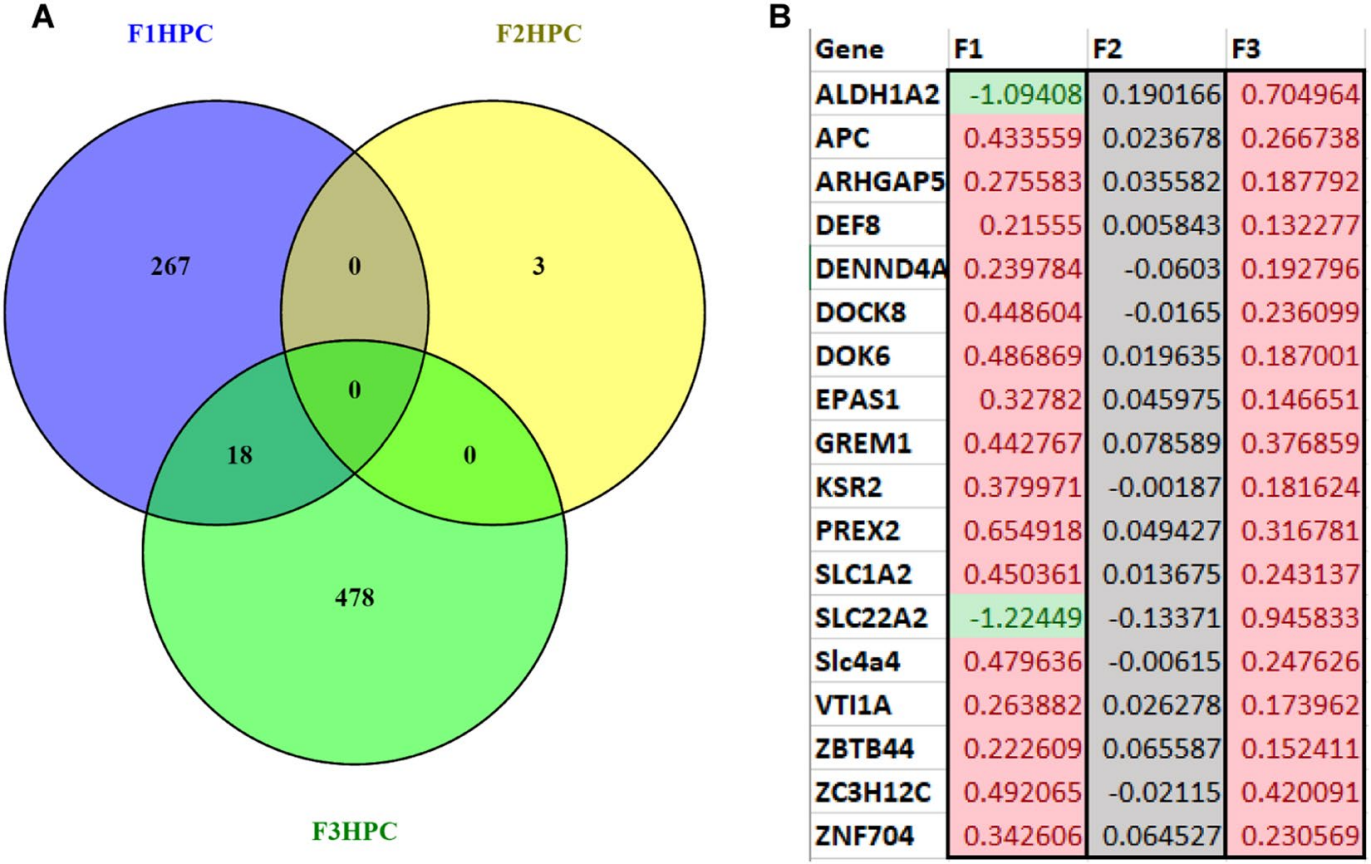
(**A**) Venn diagram illustrating the number of genes that are significantly differentially expressed in the HPC of young female F_1_-F_3_ offspring following prenatal synthetic glucocorticoid treatment of the F_0_ pregnancy and the number of genes that overlap between generations (Veh F_1_ (n = 6), F_2_ (6), F_3_ (6); sGC F_1_ (5), F_2_ (6), F_3_ (7)). (**B**) Heatmap of the 18 genes that are differentially expressed in F_1_ and F_3_ female offspring. Values indicate the log-fold change in gene expression in sGC animals relative to control, color further indicates the direction of change (green: significantly down-regulated; red: significantly up-regulated; grey: not significant).

**Figure 2:**
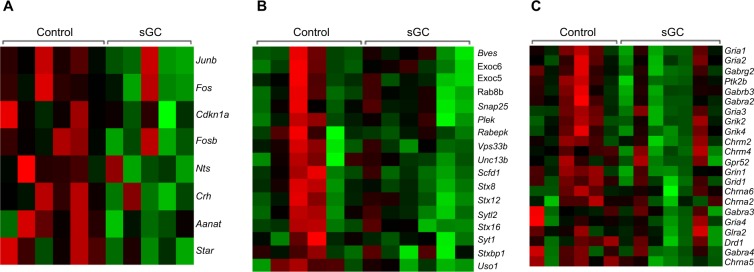
Heatmaps showing key hippocampal genes driving the gene set enrichment differences between sGC exposed offspring lineages and controls (Veh F_1_ (n = 6), F_2_ (6), F_3_ (6); sGC F_1_ (5), F_2_ (6), F_3_ (7)) in (**A**) F_1_ juvenile females, the corticosterone response pathway was significantly down-regulated (NE > 1.6, p < 0.01, FDR < 0.05); (**B**) F_2_, the vesicle docking pathway was significantly down-regulated (NES > 1.6, p < 0.01, FDR < 0.05); (**C**) F_3_, the neurotransmitter receptor activity pathway was down-regulated (NES > 1.6, p < 0.01, FDR < 0.05). Green: decreased transcription; red: increased transcription.

In F_1_, 285 genes were significantly differentially expressed in the hippocampi of sGC animals compared to control (FDR < 0.05; Fig. 1A). Of these, 69 genes are significantly down-regulated, while 216 genes are significantly up-regulated in sGC offspring (FDR < 0.05). GSEA revealed that 181 gene sets were enriched, with 146 sets negatively enriched (down-regulated) and 35 sets positively enriched (up-regulated) in sGC animals (NES > 1.6, FDR < 0.25; Supplementary Table S1). Down-regulated gene sets included hormone activity, neurotransmitter binding and corticosterone response pathways, and positively enriched gene sets included inflammatory response and locomotor behaviour pathways in the sGC animals (NES > 1.6, FDR < 0.25). An example of the corticosterone response pathways gene set is presented in Fig. 2A.

In F_2_, only three genes were significantly differentially expressed in sGC animals compared to control (FDR < 0.05; Fig. 1A); the three genes exhibited reduced expression. GSEA results showed significant enrichment of 193 gene sets, with 20 down-regulated and 172 gene sets up-regulated (NES > 1.6, FDR < 0.25; Supplementary Table S1). It is important to note that with regards to the GSEA, the individual genes within the gene sets are not significantly differentially expressed. Rather, a greater number of genes pertaining to these pathways are changed in the same direction than would be expected by chance; thus, these pathways are “enriched”^32^. Down-regulated gene sets were involved in vesicular docking and extracellular matrix organization, while up-regulated pathways were related to cytokine signaling (NES > 1.6, FDR < 0.25). An example of the vesicular docking gene set is presented in Fig. 2B.

In F_3_, 496 genes were significantly differentially expressed in sGC animals compared to control (FDR < 0.05; Fig. 1A). Of these, 223 genes were significantly down-regulated in sGC offspring, while 273 genes were significantly up-regulated in sGC offspring. Overall, there are 368 gene sets enriched as per GSEA, of which 190 are negatively enriched and 178 are positively enriched (NES > 1.6, FDR < 0.25; Supplementary Table S1). GSEA revealed significant down-regulation of neurotransmitter receptor activity and neuropeptide and synaptic signaling pathways, and up-regulation of astrocyte differentiation and Schwann cell development pathways in the sGC animals (NES > 1.6, FDR < 0.25). Figure 2C presents an example of the neurotransmitter receptor activity gene set.

### Transgenerational comparisons

There were 18 common genes that were significantly upregulated in F_1_ and F_3_ juvenile female sGC offspring compared to controls (Fig. 1B), though these 18 genes were not differentially expressed in F_2_ (Fig. 1B). There were 8 GSEA pathways that were enriched in all three generations of sGC animals (NES > 1.6, FDR < 0.25). Interestingly, the direction of enrichment of these pathways is not consistent across the generations (Table 1).

**Table 1:** Gene sets that are enriched in F1, F2, and F3 female hippocampus after antenatal sGC exposure.

Name	Type	Generation	Effect of sGC	SIZE	NES	NOM p-val	FDR q-val
GO_COLLAGEN_FIBRIL_ORGANIZATION	c5	F_1_	Negative	31	1.64	0.009	0.174
GO_COLLAGEN_FIBRIL_ORGANIZATION	c5	F_2_	Negative	31	1.92	<0.001	0.060
GO_COLLAGEN_FIBRIL_ORGANIZATION	c5	F_3_	Positive	31	−1.89	<0.001	0.056
GO_EMBRYONIC_CAMERA_TYPE_EYE_MORPHOGENESIS	c5	F_1_	Positive	18	−1.98	0.005	0.084
GO_EMBRYONIC_CAMERA_TYPE_EYE_MORPHOGENESIS	c5	F_2_	Positive	17	−1.79	0.008	0.102
GO_EMBRYONIC_CAMERA_TYPE_EYE_MORPHOGENESIS	c5	F_3_	Positive	17	−1.76	0.005	0.100
GO_EXTRACELLULAR_MATRIX_STRUCTURAL_CONSTITUENT	c5	F_1_	Negative	56	1.90	<0.001	0.015
GO_EXTRACELLULAR_MATRIX_STRUCTURAL_CONSTITUENT	c5	F_2_	Negative	56	2.02	<0.001	0.012
GO_EXTRACELLULAR_MATRIX_STRUCTURAL_CONSTITUENT	c5	F_3_	Positive	46	−1.77	0.005	0.165
GO_MULTICELLULAR_ORGANISMAL_MACROMOLECULE_METABOLIC_PROCESS	c5	F_1_	Negative	49	1.83	<0.001	0.057
GO_MULTICELLULAR_ORGANISMAL_MACROMOLECULE_METABOLIC_PROCESS	c5	F_2_	Negative	50	1.98	<0.001	0.044
GO_MULTICELLULAR_ORGANISMAL_MACROMOLECULE_METABOLIC_PROCESS	c5	F_3_	Positive	49	−2.16	<0.001	0.006
GO_NEUROPEPTIDE_SIGNALING_PATHWAY	c5	F_1_	Negative	51	1.74	0.001	0.116
GO_NEUROPEPTIDE_SIGNALING_PATHWAY	c5	F_2_	Positive	55	−1.70	0.006	0.150
GO_NEUROPEPTIDE_SIGNALING_PATHWAY	c5	F_3_	Negative	54	2.20	<0.001	0.001
REACTOME_COLLAGEN_FORMATION	c2	F_1_	Negative	48	1.96	<0.001	0.003
REACTOME_COLLAGEN_FORMATION	c2	F_2_	Negative	49	2.47	<0.001	<0.001
REACTOME_COLLAGEN_FORMATION	c2	F_3_	Positive	48	−2.15	<0.001	<0.001
REACTOME_EXTRACELLULAR_MATRIX_ORGANIZATION	c2	F_1_	Negative	58	1.98	<0.001	0.002
REACTOME_EXTRACELLULAR_MATRIX_ORGANIZATION	c2	F_2_	Negative	59	2.36	<0.001	<0.001
REACTOME_EXTRACELLULAR_MATRIX_ORGANIZATION	c2	F_3_	Positive	58	−2.26	<0.001	<0.001
GO_MULTICELLULAR_ORGANISM_METABOLIC_PROCESS	c5	F_1_	Negative	58	1.83	0.001	0.054
GO_MULTICELLULAR_ORGANISM_METABOLIC_PROCESS	c5	F_2_	Negative	60	1.77	<0.001	0.210
GO_MULTICELLULAR_ORGANISM_METABOLIC_PROCESS	c5	F_3_	Positive	58	−1.86	0.002	0.072

Type: collections of gene sets; c5 = gene ontology, c2 = curated gene sets from published data sets. Effect of sGC: describes relationship between treatment with sGC and the expression of genes within a gene set, positive-increased expression, negative-decreased expression. Size: number of genes in the gene set. NES: normalized enrichment score used to compare gene sets of different sizes. FDR: false discovery rate, the probability that a gene set with a specific NES is a false positive. (Veh F_1_ (n = 6), F_2_ (6), F_3_ (6); sGC F_1_ (5), F_2_ (6), F_3_ (7)).

### DNA methylation

**Figure 3:**
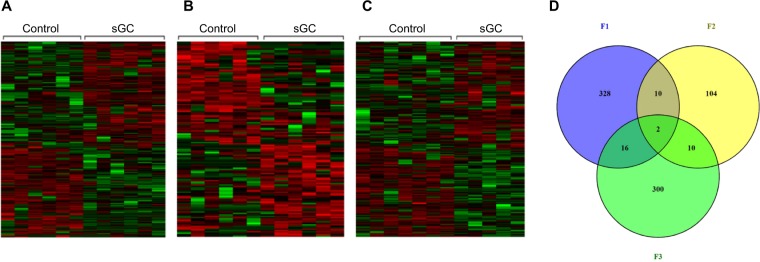
Heatmaps showing significantly differentially methylated regions (DMRs) in the hippocampus between sGC exposed offspring lineages and controls (Veh F_1_ (n = 6), F_2_ (6), F_3_ (7); sGC F_1_ (6), F_2_ (6), F_3_ (5)) in (**A**) F_1_ juvenile females, a total of 406 regions were differentially methylated; 184 demethylated, 222 hypermethylated (>10%, FDR < 0.05); (**B**) In F_2_, 139 regions were differentially methylated; 74 demethylated, 65 hypermethylated (>10%, FDR < 0.05); (**C**) In F_3_, 380 regions were differentially methylated; 170 demethylated, 210 hypermethylated (>10%, FDR < 0.05). Green decreased methylation, red increased methylation; (**D**) Venn diagram illustrating the number of regions related to genes that are significantly differentially methylated in the HPC from F_1_-F_3_ female PT (Paternal Transmission) and the number of regions that overlap between generations.

In F_1,_ there were 406 significantly differentially methylated regions (DMRs; 100 bp window) (>10%, FDR < 0.05) with 184 DMRs hypomethylated (>10%, FDR < 0.05) and 222 DMRs hypermethylated (>10%, FDR < 0.05) in sGC offspring relative to control (Fig. 3A, Supplementary Table S2). Hypomethylated DMRs were overrepresented in 14 pathways (p < 0.01, FDR < 0.05) related to ion and glutamate channel activity, while hypermethylated DMRs were overrepresented in 54 pathways (p < 0.01, FDR < 0.05), related to nervous system development and synapse assembly (Supplementary Table S3). In F_2_, there were 139 significant DMRs (>10%, FDR < 0.05) with 74 DMRs hypomethylated (>10%, FDR < 0.05) and 65 DMRs hypermethylated (>10%, FDR < 0.05) in sGC animals relative to control (Fig. 3B, Supplementary Table S2). Hypomethlyated DMRs were enriched in 7 pathways (p < 0.01, FDR < 0.05) related to GTPase binding and regulator activity, while hypermethylated DMRs were only enriched in the cytokine receptor binding pathway (p < 0.01, FDR < 0.05), related to nervous system development and synapse assembly (Supplementary Table S3). In F_3,_ there were 380 significant DMRs (> 10%, FDR < 0.05) with 170 DMRs hypomethylated (>10%, FDR < 0.05) involved in glutamatergic and ion channel activity and 210 DMRs hypermethylated related to acetylcholine receptor binding and neuron recognition (>10%, FDR < 0.05) in sGC animals relative to controls (Fig. 3C, Supplementary Table S2).

Not all of the DMRs significantly affected by sGC exposure were associated with coding genes (Supplementary Table S2). Figure 3D shows the number of hypo- or hypermethylated DMRs in each generation of offspring that were related to genes. There were 2 genes with differential methylation in all three generations of sGC offspring. These genes are *Kif26b*, and *St6galnac5* (>10%, FDR < 0.05; Fig. 3D). Methylation of *Kif26b* is decreased in F_1_ and F_2_ and increased in F_3_, whereas methylation of *St6galnac5* is decreased in F_1_ and F_3_ and increased in F_2_.

### Overlap between changes in DNA methylation and gene expression

**Figure 4:**
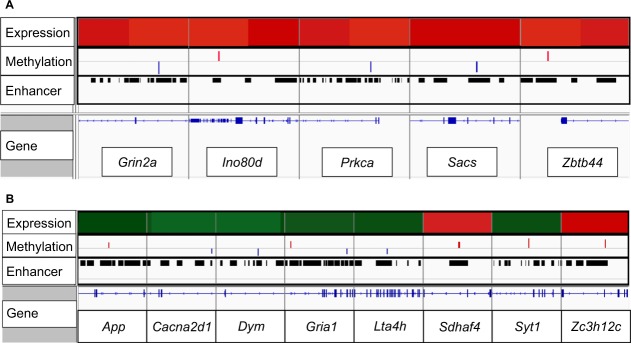
Visualization of significant differences in expression and DNA methylation, overlaid with genomic loci in (**A**) F_1_ and (**B**) F_3_. Top row represents significant changes in gene expression associated with prenatal sGC. Red indicates significantly increased expression (FDR < 0.05), green indicates significantly decreased expression (FDR < 0.05). Second row represents significant changes in methylation. Blue indicates significantly decreased methylation (>10%, FDR < 0.05), red indicates significantly increased methylation (>10%, FDR < 0.05). Enhancer row identifies which regions of the genome were captured as enhancers. Gene tracks are represented above the gene names in blue, with horizontal lines representing intron sequences and coding exons represented by blocks.

In F_1_, there were 5 genes (*Ino80d, Zbtb44, Grin2a, Sacs, Prkca*) that were both significantly differentially expressed (FDR < 0.05) and differentially methylated (>10%, FDR < 0.05; Fig. 4A). There was no overlap between gene expression and methylation in F_2_. In F_3_, 8 genes (*App, Cacna2d1, Dym, Gria1, Lta4h, Sdhaf4, Syt1, Zc3h12c*) were both significantly differentially expressed (FDR < 0.05) and differentially methylated (>10%, FDR < 0.05; Fig. 4B). All the methylation changes that overlapped gene expression in both F_1_ and F_3_ animals occurred in enhancer regions (Fig. 4).

### Single-nucleotide resolution methylation changes are related to small non-coding RNAs (snRNAs)

To elucidate how antenatal sGC affect DNA methylation at the individual CpG level, we performed DNA methylation analysis with single-nucleotide resolution. In F_1_ animals that had been exposed to prenatal sGC, there were 63 CpGs that were significantly differentially methylated (>10%, FDR < 0.05; 29 hypomethylated, 34 hypermethylated). There were 13 differentially methylated CpGs in unannotated regions, 5 were in regions related to coding genes, and 45 related to snRNA encoding genes. In F_2_ sGC animals, there were 21 CpGs that were significantly differentially methylated (>10%, FDR < 0.05; 11 hypomethylated, 10 hypermethylated). There were 4 differentially methylated CpGs in unannotated regions, 4 were in regions related to coding genes and 13 were related to snRNA genes. In F_3_ sGC animals, there were 51 CpGs that were significantly differentially methylated (>10%, FDR < 0.05; 33 hypomethylated, 18 hypermethylated). There were 12 differentially methylated CpGs in unannotated regions, 8 in regions related to coding genes, and 31 were related to snRNA genes. The significantly differentially methylated CpGs were distributed amongst promoter and enhancer regions. A list of affected snRNA genes is presented in Supplementary Table S4.

### sGC differentially methylated CpGs were enriched in RNApol II-PS5 binding regions

**Figure 5:**
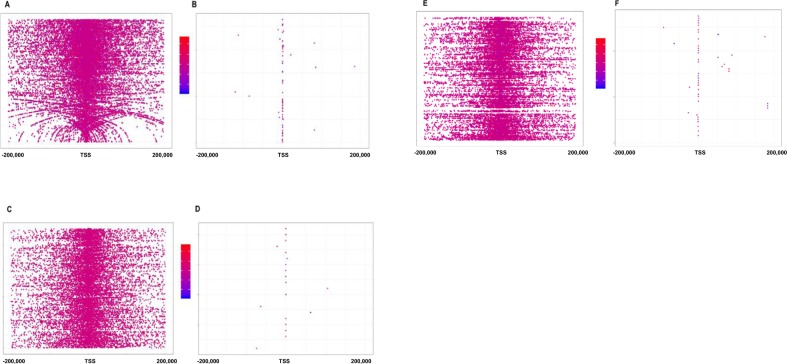
CpG distributions with respect to transcription start sites (TSS) in (**A**) F_1_ distribution of all CpG sites captured with respect to distance to TSS. (**B**) Distribution of significantly differentially methylated CpGs in F_1_ (p < 0.05, FDR < 0.05) with respect to distance to TSS. (**C**) F_2_ distribution of all CpG sites captured with respect to distance to TSS. (**D**) Distribution of significantly differentially methylated CpGs in F_2_ (p < 0.05, FDR < 0.05) with respect to distance to TSS. (**E**) F_3_ distribution of all CpG sites captured with respect to distance to TSS. (**F**) Distribution of significantly differentially methylated CpGs in F_3_ (p < 0.05, FDR < 0.05) with respect to distance to TSS. Red indicates increased methylation, while blue indicates decreased methylation. Veh F_1_ (n = 6), F_2_ (6), F_3_ (7); sGC F_1_ (6), F_2_ (6), F_3_ (5).

We investigated the enrichment of significantly differentially methylated CpGs among distinct genomic features. Since we designed a custom probe set to target specific regions of the genome, we were able to use this probe coverage to determine whether methylation changes were enriched in a certain region. Though over 85% of our capture was designed for enhancer regions, the significant changes in CpG methylation did not occur in enhancer regions, but rather occurred in promoter regions. The observed distribution of differentially methylated CpGs (which occurred in the promoter regions) was significantly different from the expected distribution generated by the custom probe capture design (which captured more enhancer regions than promoter regions) in F_1_ (*χ*^2^ = 155.46, p = 2.2e-16), F_2_ (*χ*^2^ = 20.26, p = 4.4e-04), and F_3_ (*χ*^2^ = 77.42, p = 2.9e-15). In all three generations, differentially methylated CpGs occurred in promoter/RNAPol II-PS5 binding regions; F_1_ (*χ*^2^ = 562.71, p < 2.2e-16), F_2_ (*χ*^2^ = 16.51, p = 4.8e-05), and F_3_ (*χ*^2^ = 20078, p < 2.9e-16) (Fig. 5).

### Differential expression of candidate genes

Since many studies have shown that antenatal sGC exposure alters gene expression in the hippocampus, we used these findings to generate a list of genes (Supplementary Table S5) that we hypothesized would be most affected with regards to gene expression and DNA methylation as a result of antenatal sGC exposure. In the F_1_ sGC offspring, five genes from our *a priori* list (*Kcnj6, Myo5a, Crhr2, Grin2a, Ncoa2)* were differentially expressed. To test our hypothesis that the genes from our *a priori* list were most affected by antenatal sGC, we performed *χ*^2^ to determine whether more genes from our *a priori* list were significantly differentially expressed than would be expected by chance. Contrary to our hypothesis, the number of observed genes was not significantly different than would be expected by chance (*χ*^2^ = 0.0037, p = 0.95). There were no genes differentially expressed from the *a priori* list in F_2_. In the F_3_ sGC offspring, 16 genes from the *a priori* list were significantly differentially expressed (*Hmgb*2*, Shank1, Emp2, Gpm6b, Ssr4, Chd3, Gldc, Dlg4, Atp6v1c1, Gria1, Nr3c2, Snap25, Gad2, Dync1l1, Syp, Gad1)*, more than would be expected by chance (*χ*^*2*^ = 7.13, p < 0.05).

### Methylation of genes of interest

To test our hypothesis that the genes from our *a priori* list were most affected by antenatal sGC, we performed *χ*^2^ to determine whether more genes from our *a priori* list were significantly differentially methylated than would be expected by chance. 11 genes (*Kcng1, Mbd*2*, Grin*2*a, Snap25, Adrb2, Gria4, Prmt5, Mecp2, Nr3c1, Epb41l2, Hdac2*) from the *a priori* list were significantly differentially methylated in F_1_ offspring (>10%; FDR < 0.05) which is not more than would be expected by chance (χ^2^ = 1.76, p = 0.18). In F2, eight genes from the list were significantly differentially methylated (*Iqgap2, Gatad2a, Kit, Camk2a, Bdnf, Grik1, Fbxl20, Camk2d*; >10%; FDR < 0.05), which is not more than would be expected by chance (*χ*^*2*^ = 2.74, p = 0.10). In F_3_, 6 genes from the *a priori* list were significantly differentially methylated (*Camk2d, Camk2b, Gria1, Grid2, Cacna1b, Grin2b*; > 10%; FDR < 0.05), also not more than expected by chance (*χ*^*2*^ = 0.36, p = 0.55).

## Discussion

We have demonstrated, for the first time, that transgenerational changes in hippocampal gene expression and methylation occur over three generations in female offspring following antenatal sGC and paternal transmission. Strikingly, while only 3 genes were differentially expressed in the F_2_ sGC offspring, there were over 280 genes differentially expressed in F_1_ sGC offspring, and over 490 genes were differentially expressed in F_3_ sGC offspring. There was no overlap between differentially expressed genes across all 3 generations. Furthermore, we observe generation-specific hippocampal methylation signatures in animals from the sGC-exposed lineage. Importantly, changes in methylation that are associated with changes in gene expression occur at enhancer regions, and methylation changes at the individual CpG level are enriched in RNApol II-PS5 binding regions of small non-coding RNAs (snRNAs). Thus, antenatal sGC exposure results in generation-specific methylation signatures in the hippocampus, which may function in concert with transcriptional machinery to alter phenotypes and transmit effects to subsequent generations.

In F_1_ offspring exposed to prenatal sGC, 285 genes were significantly differentially expressed in the hippocampus. Previous research has shown that the expression of genes related to key signaling pathways are altered by prenatal exposures to excess glucocorticoids^6,10–14^. In the present study, using an *in silico* approach, we identified 199 genes linked to key hippocampal signaling pathways that related to published phenotypic outcomes in offspring following prenatal sGC exposure or maternal stress in pregnancy. At an individual gene level, only five genes from the *a priori* list (*Kcnj6, Myo5a, Crhr2, Grin2a, Ncoa2)* were differentially expressed in the hippocampus of F_1_ offspring following prenatal exposure to sGC. Notwithstanding, we did observe down-regulation of gene set pathways related to the corticosteroid response. The hippocampus exerts inhibitory control over the PVN^33^, thus down-regulation of genes related to corticosteroid response (50 days after sGC exposure) may result in decreased inhibition of the PVN, and an increased HPA-response to stress. This would be consistent with the increased HPA-response observed in the F_1_ sGC offspring after exposure to the open-field^21^.

Unexpectedly, we observed changes in the hippocampal expression of only three genes in F_2_ female offspring in the sGC group (paternal transmission; F_1_ fathers exposed to sGC when F_0_ grandmothers treated during pregnancy) compared to controls. Interestingly, these animals showed the strongest behavioral phenotype (of the three generations), as well as substantial changes in gene expression in the hypothalamic paraventricular nucleus, and prefrontal cortex^21,26^. While individual genes may not have been significantly differentially expressed, we used gene set enrichment analysis, which examines whether there are small changes in gene expression in the same direction (up or down) for multiple genes related to the same pathway, to identify gene networks that are differentially regulated as a result of antenatal sGC. We observed that pathways involved in vesicular docking are down-regulated in the F_2_ descendants of sGC-exposed pregnancies. In addition, pathways involved in blood-brain barrier integrity (collagen and extracellular matrix) were down-regulated in the F_2_ sGC offspring hippocampus. Similar gene sets have previously been shown to be altered by antenatal glucocorticoids in the PVN^21,34^. Thus, very few individual genes are significantly differentially expressed in the F_2_ sGC offspring, however, dysregulated gene networks may suggest altered hippocampus gene function in these animals.

In F_3_ offspring, descendent of sGC treated mothers, 16 genes from the *a priori* list were significantly differentially expressed, which was more than was expected by chance. Furthermore, while the effects of antenatal sGC decrease in the F_3_ compared to F_1_ offspring in the PVN and PFC^21,26^ in the present study, we demonstrated that in the hippocampus, more genes are significantly affected by sGC in the F_3_ animals compared to the F_1_ and F_2_ offspring. Interestingly, we observed downregulation of *Mr* (*Nr3c2*), in the F_3_ sGC exposed animals. *Mr* expression in the hippocampus plays an important role in HPA negative feedback regulation^35^, and decreased *Mr* signaling may be indicative of decreased HPA-inhibition. However, the F_3_ sGC offspring did not display altered HPA response to stress, though they did demonstrate a hyperactive phenotype in the open-field^21^. These findings suggest that glucocorticoid signaling may be altered in the hippocampus of F_3_ sGC animals and may relate to phenotypic outcomes. In the present study, we also observed down regulation of genes that are important for learning and memory (*Gad1, Syp, Gad2, Gria1*). *Gad1* and *Gad2* are enzymes involved in γ-aminobutyric acid (GABA) signaling^36^, and decreased expression of these genes in the hippocampus has previously been observed in patients with schizophrenia and bipolar disorder^37^. *Syp* is involved in regulating activity-dependent synapse formation^38^, and *Gria1* expression is essential in new memory formation^39,40^. Though behavioral tests related to cognition were not performed in the present study, the gene expression changes observed in F_3_ sGC offspring suggest these animals may have had learning deficits, and this can be tested in future studies.

We observed generation-specific effects of antenatal sGC exposure, with distinct methylation signatures occurring in each of the generations. We observed very little overlap between gene expression and methylation changes. We have previously shown that transcriptionally active enhancers are less methylated than *poised* enhancers^31^. However, the mRNA measure of transcription level is confounded by several regulatory processes downstream to transcription initiation^41^, meaning that enhancer methylation and mRNA content do not always correlate. This may be why there were so few changes in DNA methylation that correlate with steady-state expression. However, changes in methylation that are associated with changes in steady-state gene expression did occur in enhancer regions, consistent with the notion that DNA methylation in enhancer regions is more correlated with gene expression than promoter methylation^24,30^. Epigenetic mechanisms play a role in regulating gene expression patterns in response to environmental cues^42^. The animals in this study were euthanized in an unstressed basal state, and methylation signatures left by antenatal sGC may indicate that genes are poised to be expressed differently following environmental stimuli. It may be that stronger correlations between gene expression and methylation would occur when the animals are in an activated state (*i.e*. a stressful environment). Interestingly, very few genes from our *a priori* list displayed significant changes in methylation. Though previous research has demonstrated that antenatal sGC alters the methylation status of promoter regions of genes from this list^6^, these changes have also been shown to be dynamic, and may not remain as a permanent signature 50-days after final exposure to sGC^6^. Only two genes were differentially methylated across all three generations of offspring: *Kif26b*, a motor protein involved in organelle transport and intracellular signaling^43^; and *St6galnac5*, a glycosyltransferase involved in cell-cell interactions^44^. Both genes demonstrated generation-specific changes in methylation but were not differentially expressed due to sGC exposure. Unlike our previous work in the PFC^26^, we did not observe consistent changes in DNA methylation and gene expression in the hippocampus, suggesting region-specific responses following prenatal sGC exposure.

Analyzing DMRs (100 bp windows of DNA methylation changes) provides an overview of the methylation signatures that result from antenatal sGC exposure. In the F_1_ and F_3_ sGC offspring, hypomethylated DMRs were related to glutamatergic signaling. While glutamate release is essential for cellular signaling processes, including learning and memory, the hippocampus is especially vulnerable to excitotoxicity from excess glutamate release. Chronic stress, and chronic exposure to glucocorticoids, is known to be a potent trigger of glutamate excitotoxicity, and results in cell death and mood disorders^45^. Corticosteroids potentiate the excitability of the hippocampus^46^ and prime the hippocampal circuit for subsequent stimulation^47^. Corticosteroids have been shown to result in a rapid release of glutamate^46^ and increased glutamatergic synaptic signaling has been shown to coincide with decreased levels of methylation^48,49^. Taken together, these findings suggest that the hypomethylation observed in the F_1_ and F_3_ sGC offspring is indicative of altered glutamatergic sensitivity, which may lead to decreased excitotoxicity resilience in the hippocampi of these animals. Surprisingly, this DNA methylation signature was not present in the F_2_ sGC animals. In the F_2_ sGC animals, hypomethylation was observed in genes related to GTP activity which is an essential part of G-coupled protein receptor signaling^50^. Rab GTPase activity has been shown to be involved in signal transduction of glutamate receptors^51^, thus it is possible that glutamate receptor activity may also be altered in F_2_ sGC animals, but *via* a different mechanism.

The significantly differentially methylated CpGs in all three generations of sGC offspring were enriched in RNApol II-PS5 binding sites of small noncoding nucleolar and spliceosomal RNA genes. Small noncoding RNAs (snRNAs) are a secondary level of epigenetic control involved in the fine-tuning of gene expression^52,53^. Promoter methylation levels have been shown to regulate snRNA expression^54^, and expression of snRNAs has been shown to be dysregulated in the adult mouse brain after fetal alcohol exposure, which may suggest that altered snRNA expression can influence phenotype resulting from early life exposure^52^. In the current study, mRNA-enriched analyses were performed to quantify gene expression, which precludes us from assessing changes in the expression of snRNA and represents a limitation of this study. However, we observed differential methylation for snRNAs (*Snora2*) responsible for post-transcriptional modifications of spliceosomal RNA (*U6*) across all three generations of sGC offspring. *U6* is involved in the removal of intronic regions of RNA primary transcript and assembly of exons to form mRNA^55^. Though the exact mechanisms remain to be elucidated, recent evidence suggests that altered snRNA activity is involved in alternative splicing^55^. Alternative splicing, a conserved process that increases the diversity of the transcriptome and proteome by allowing multiple mRNA products to result from a single gene, has been shown to be regulated by interplay between chromatin and DNA methylation^56–58^. Over 90% of human genes undergo alternative splicing^58^, and alternative splicing patterns have been shown to be sex-specific^59^, and heritable in a Mendelian fashion^60^. Thus, antenatal sGC results in transgenerational changes in DNA methylation in RNApol II-PS5 binding regions of snRNA genes involved in transcription machinery, implicating alternative splicing as a potential mechanism involved in the transgenerational transmission of the effects of antenatal sGC in the hippocampus. However, this important possibility needs to be tested in further detailed experiments.

In the present study, the fact that there are behavioural changes accompanied by changes in hippocampal DNA methylation and gene expression across three generations implicates male germ-line epigenetic transmission. A number of potential mechanisms, both direct and indirect, by which this might occur are emerging. Persistent changes in germline DNA methylation, as a mode of transgenerational transmission, are unlikely given the broad waves of DNA demethylation that occur during embryonic development. In support of this, a previous study has shown maternal malnutrition to be associated with differentially methylated regions within the sperm of F_1_ offspring, but that no differences in methylation were maintained into the F_2_ generation^61^. Another potential route of paternal transmission may be through small RNAs, including microRNA (miRNA) and transfer RNA. Sperm contain miRNA which can be delivered to the oocyte on fertilization^62^. Early life stress in males, resulted in an altered compliment of miRNA in sperm in adulthood, and an elegant series of subsequent studies determined that a number of these miRNA were driving phenotypic differences in subsequent offspring^62,63^. Other studies have indicated that histone modifications in sperm as a potential route for paternal inheritance. While the majority of histones in sperm are replaced by protamines, some histones remain^64^. After fertilization, paternal protamines are replaced by heavily acetylated maternal histones, while paternal histones remain largely untouched^65^. Thus, it is possible for epigenetic marks on these histones to be inherited.

Epigenetic inheritance paradigms have often considered potential mechanisms in isolation; however, it is likely that there is interplay between processes. In this regard, small RNAs can drive *de novo* cytosine methylation and alterations in chromatin structure^66^. Such integrated processes may be occurring in the model described in the present study, allowing the transmission of the effects of sGC exposure across multiple generations. The dynamic nature of the changes across 3 generations suggest that an initial epigenetic signal transmitted by the F_1_ sperm is not a static epigenetic memory that is inherited across future generations. The picture that is emerging is more consistent with the initial epigenetic signal triggering a cascade of epigenetic events that include DNA methylation which keep changing dynamically across tissues and generations. Clearly further studies are required to determine the specific processes involved in this model.

There are some limitations with the present study. Focus was placed on DNA methylation and gene transcription and future studies should address the relationship of altered gene transcription with altered protein levels in the hippocampi across multiple generations. Analysis of DNA methylation was limited to 5-methylcytosine. We did not undertake analysis of downstream modifications by oxidation of 5 methylcytosine such as 5-hydroxymethylcytosine, 5-formylcytosine and 5-carboxylcytosine, which may also play a role in transgenerational transmission. We also acknowledge that our use of RNAPolII-Ser5 ChIP to assist in the identification of promoters may lead to some biases towards active rather than silenced genes. Our studies have highlighted the possibility that prenatal sGC may lead to alterations in transcript splicing. Due to the sequencing methodology used in this study, we were unable to investigate differential splicing, though this could be a focus of future studies. Due to budgetary constraints, analysis was limited to female juvenile offspring (where greatest phenotypes were observed). Future comparative analyses in males would certainly provide insight into the relationship between alterations in methylation, gene expression and phenotype following antenatal sGC exposure, as well as sex differences in the molecular actions of prenatal sGC exposure in the developing hippocampus. Finally, future studies are required to further elaborate the mechanisms involved in transgenerational transmission.

## Conclusions

This study demonstrates transgenerational changes in transcription and DNA methylation following antenatal sGC exposure over the paternal lineage. Changes in gene transcription and DNA methylation patterns following antenatal sGC are generation-specific and are highest in the third-generation offspring. DNA methylation changes associated with sGC exposure may be involved in altered glutamatergic signaling. Significant changes in individual CpG methylation occur in RNApol II-PS5 binding regions of snRNAs and may implicate alternative splicing as a mechanism involved in transgenerational transmission of the effects of antenatal sGC. These findings demonstrate that the effects of antenatal sGC exposure alter genetic and transcriptomic regulation of the hippocampus of three generations of offspring through the paternal lineage. These findings provide new perspectives on the mechanisms involved in transgenerational transmission and show that the effects of antenatal sGC on the hippocampus may potentiate with advancing generations. Thus, it is imperative to perform future studies in human cohorts to elucidate the long-term effects of antenatal sGC on the developing brain and identify interventions to prevent transmission to subsequent generations.

## Methods

### Animals

Twelve-week-old Dunkin-Hartley guinea pigs (F_0_: Charles River, St Constant, QC, Canada) were mated as previously described^21^. Pregnant guinea pigs received 3 courses of the sGC betamethasone (sGC; 1 mg/kg; Betaject phosphate-acetate mix; Sabex Boucherville, QC, Canada) or saline (0.166 ml/kg) on gestational days (GD) 40 & 41, 50 & 51 and 60 & 61, as outlined previously^21^. Delivery in Dunkin-Hartley guinea pigs occurs at ~69 days, with an average of 3 offspring/litter in our colony. The sGC dose utilized in the present study is comparable to that given in pregnancies at risk of preterm delivery (~0.25 mg/kg), as the guinea pig glucocorticoid receptor (GR) has a 4-fold lower affinity for sGC^67^. While single course treatment with GC is currently standard of care, in the late 90’s and early 2000’s multiple course therapy was widespread^68^, and more recently the use of repeat ‘rescue’ sGC treatment has been adopted^69^.

First (F_1_) generation male offspring, derived from independent mothers, were mated with non-experimental females (purchased from Charles River) to generate F_2_ offspring, as previously described^21^. Third (F_3_) generation offspring were generated by mating the F_2_ males with non-experimental females. The males and non-experimental females were only bred once. Other than routine cage maintenance, F_1_ and F_2_ pregnancies were left undisturbed. A figure outlining the full breeding regimen has been published^21^. As we have reported previously, there was no significant effect of prenatal sGC treatment on breeding parameters in any of the generations, including litter size and sex ratio^21^. All protocols were approved by the Animal Care Committee at the University of Toronto in accordance with the Canadian Council on Animal Care.

Animals were euthanized in an unstressed basal state on post-natal day 40, as previously reported^21^, and both left and right hippocampi were removed and frozen immediately on dry ice. To analyze gene expression and DNA methylation from these tissues, genomic DNA and RNA were extracted simultaneously from 20 mg of powdered right hippocampus using the AllPrep DNA/RNA/miRNA Universal Kit (Qiagen, Ontario, Canada). In the present study, the hippocampi from female juvenile offspring (n = 5–7/gp; F_1_, F_2_, F_3_ vehicle; F_1_, F_2_, F_3_ sGC) were used for molecular analysis. Our previous studies had indicated that juvenile females showed the greatest differences in behavioural and neuroendocrine phenotypes (pituitary-adrenal function and open-filed activity) associated with prenatal sGC exposure^21^. All female offspring used in this study were derived from independent mothers, and each mother had been bred to a single F_1_ or F_2_ male.

### Custom design of capture arrays for bisulfite mapping of DNA methylation

We designed a custom targeted array (SeqCap Epi Enrichment, Roche), based on our previous study^31^, composed of enhancer and promoter regions of the guinea pig genome. Promoter regions were identified from the Ensemble database, as well as from a previous RNAPolII-Ser5 ChIP experiment, the serine 5 phosphorylation marks RNA polymerase 2 molecules engaged in turning on transcription^31^. Enhancer regions were identified in a previous H3K4me1 ChIP experiment, H3K4me1 mark is enriched at enhancers^31^. The SeqCap probe enrichment kit from Roche allowed for the capture of 210 Mb of gDNA. 550 bp of promoter regions centered around the TSS were covered for all genes (26129 promoters equating to 6.8% of the capture). RNA PolII-Ser5 peaks were captured, along with RNA PolII-Ser5 peaks that coincided with H3K4me1 peaks (66909 regions; 13% of capture). Enhancer peaks smaller than 550 base pairs were captured in their entirety (49866 enhancers, equating to 8.5% of capture) and 550 bp of larger enhancer peaks were captured to span the center of the peak (263249 enhancers; 69% of capture). Lastly, a list of gene networks that we hypothesized would be most affected by sGC, based on previous studies^6,10–14^ was generated using 14 ‘seed’ genes (*Grin2b*, *Nr3c2*, *Nr3c1*, G*ad1*, *Drd1, Crh, Abcb1, Gria1, Sert, Dnmt1, Fos, Bdnf, Syp, Mbd2*) for which the top 20 genes related to biological process were selected^61^. This resulted in a list of 199 genes of interest (Supplementary Table S5). Probes were designed to cover all promoters and enhancers for the genes of interest (1260 enhancers; 2% of capture).

### Capture, bisulfite conversion

gDNA (1 µg; F_1_: Veh N = 6, sGC N = 6; F_2_: Veh N = 6, sGC N = 6; F_3_: Veh N = 7, sGC N = 5) was purified using the AMPure XP beads (Beckman Coulter, Ontario, Canada), following the manufacturer’s instructions (1.8 v/v). Libraries were prepared from purified DNA, using the KAPA Library Prep Kit Illumina (Roche) and SeqCap Adapter kit (Roche) according to the SeqCap Epi Enrichment System User Guide. Libraries then underwent bisulfite conversion. Bisulfite conversion was performed using the EZ DNA Methylation-Gold Kit (Zymo Research), and bisulfite converted DNA was amplified using LM-PCR by a 15-cycle PCR and purified with AMPure XP beads (1 v/v) (Beckman Coulter). Size and quantity of the resulting libraries were verified using HSdna Bioanalyzer chip (Agilent, CA, USA) and Q-PCR, respectively (Kappa Library Quantification kit for Illumina sequencing). Bisulfite libraries were hybridized with our custom SeqCap Epi Probe pool. After washing and recovery of captured DNA, an amplification using LM-PCR was performed as described above. All captured samples were sequenced by 50 bp pair end-sequencing Illumina HiSeq. 2000 sequencing at Institut de Recherches Cliniques de Montreal (IRCM).

### Methylation capture sequencing analyses

After bisulfite treatment and sequencing, reads were trimmed using Trimmomatic-0.32, then aligned to the guinea pig genome (cavPor3) using bsmap-2.74 v0.12.5. Picard-tools-1.93 was used to remove duplicates. Sequenced reads showed on average 68% on-target alignment with capture-probe design. Methylation levels were determined for individual CpG sites using bsmap-2.74 with a minimal coverage of 10 reads. Changes in methylation for 100 bp windows (50 bp apart) and individual CpGs were detected using the calculate DiffMeth function from MethylKit (v.1.4.0) in R (version 3.2.3). Data were annotated using Homer v4.6 with the annotatePeaks script and CavPor3 genome.

### RNA sequencing

RNA quality was determined by Bioanalyzer (RNA 6000 Pico LabChip, Applied Biosystems, Ontario, Canada); all RNA (1 μg) samples RIN ≥ 7. mRNA library preparation was performed using Illumina TruSeq V2 mRNA enrichment using standard protocols. High-throughput sequencing was performed on an Illumina HiSeq. 2500 sequencing system using standard run, following the protocol recommended by Illumina for sequencing mRNA samples. Sequencing was done for each biological replicate (F_1_: Veh N = 6, sGC N = 5; F_2_: Veh N = 6, sGC N = 6; F_3_: Veh N = 6, sGC N = 7) at 1 × 51 bp by the Donnelly Centre for Cellular and Biomolecular Research (University of Toronto, Ontario, Canada). RNA-seq results were analyzed as previously described^21^. Briefly, differential gene expression was assessed using EdgeR’s (version 3.12.1)^70,71^, general linear model likelihood ratio test and FDR-corrected *p* < 0.05 was considered significant. Genotype permutations (1000) were computed in Broad Institute’s Gene Set Enrichment Analysis (GSEA)^32,72^ to determine FDR, nominal p-value, and normalized enrichment score (NES) of each gene set. Gene sets with FDR ≤ 0.25, p ≤ 0.01, and NES ≥ 1.6 met significance thresholds^21^. All sequencing data can be accessed at GEO with accession number GSE109765.

### Differentially methylated CpG distribution statistics test

The CpGs with minimum 10X coverage were categorized into ‘Exon’, ‘Intergenic’, ‘Intron’, ‘Promoter-TSS’, and ‘TTS’ (Transcription Termination Site) based on Homer v4.6 using the annotatePeaks script. The expected counts were calculated with the number of CpGs that were sequenced with 10X coverage. Statistics were calculated using multinomial goodness-of-fit Chi-square test (R; version 3.2.3). Post-hoc Chi-square tests were run on each category (genomic region and capture design region) versus the sum of all other categories to determine which category was driving the effect.

### Gene expression (qRT-PCR)

RNA (400 ng) was converted to cDNA using SensiFAST cDNA synthesis kit (Bioline, London, England) as per the manufacturer’s instructions. The reaction included random hexamer primers and anchored oligo dT to ensure unbiased 3′ and 5′ coverage and reverse transcription of all regions. qRT-PCR was run using the SensiFAST SYBER Hi-ROX kit (20 μl reaction, Bioline) with forward and reverse primers according to the manufacturer’s instructions. qRT-PCR was performed in a Bio Rad C1000 Thermal Cycler and quantified by a CFX96 Real-Time PCR Detection System using the following conditions: 95 °C for 30 sec; followed by 40 cycles of 95 °C for 5 s, and 60 °C for 5 s, for plate read. All samples were run in triplicate. Relative expression of target mRNA (*Mineralocorticoid Receptor* (*Nr3c2), Glutamate Ionotropic Receptor NMDA type subunit 2* *A (Grin2a), Glutamate Decarboxylase 1 (Gad1), Synaptophysin (Syp)*) was normalized to *Gapdh* (see Supplementary Table S6 for primer sequences) by the 2^−ΔΔc(t)^ method. qPCR validation correlated 99% with RNAseq findings (Supplementary Fig. S5).

## Supplementary information

Supplementary Figure S1

Supplementary Table S1

Supplementary Table S2

Supplementary Table S3

Supplementary Table S4

Supplementary Table S5

Supplementary Table S6

## Data Availability

The datasets generated during and/or analysed during the current study are available from the corresponding author on reasonable request.
