# Supplementary material for: Prenatal Glucocorticoid Exposure Results in Changes in Gene Transcription and DNA Methylation in the Female Juvenile Guinea Pig Hippocampus Across Three Generations
Source: Sci Rep. 2019 Dec 3;9:18211. doi: 10.1038/s41598-019-54456-9 (PMC6890750; doi:10.1038/s41598-019-54456-9)

Prenatal glucocorticoid exposure results in changes in gene transcription and DNA methylation in the female juvenile guinea pig hippocampus across three generations

Andrea Constantinof, Lisa Boureau, Vasilis G. Moisiadis, Alisa Kostaki, Moshe Szyf, Stephen G. Matthews

Supplementary Figure S1: qRT-PCR validation of RNA-seq results. Correlation of sequencing data to qPCR data (each point represents one gene): R2=-0.9995; y=1.0927x – 0.0353

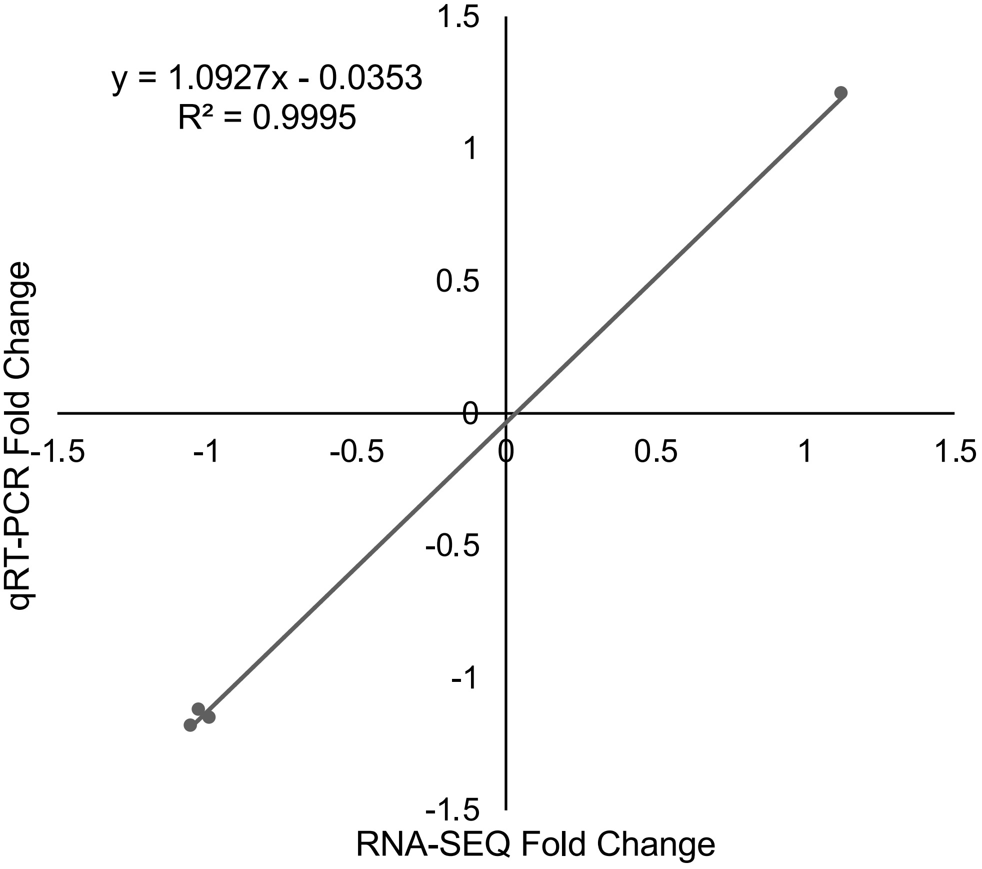

Supplement: Supplementary file 1 — Supplementary Figure S1 [file 41598_2019_54456_MOESM1_ESM.pdf]
